# Cryo-annealing of Photoreduced CdS Quantum Dot–Nitrogenase MoFe Protein Complexes Reveals the Kinetic Stability of the E_4_(2N2H) Intermediate

**DOI:** 10.1021/jacs.3c06832

**Published:** 2023-09-20

**Authors:** Gregory E. Vansuch, David W. Mulder, Bryant Chica, Jesse L. Ruzicka, Zhi-Yong Yang, Lauren M. Pellows, Mark A. Willis, Katherine A. Brown, Lance C. Seefeldt, John W. Peters, Gordana Dukovic, Paul W. King

**Affiliations:** † Biosciences Center, National Renewable Energy Laboratory, Golden, Colorado 80401, United States; ⊗ Department of Chemistry, University of Colorado Boulder, Boulder, Colorado 80309, United States; § Department of Chemistry and Biochemistry, Utah State University, Logan, Utah 84322, United States; ∥ Institute of Biological Chemistry, Washington State University, Pullman, Washington 99163, United States; ⊥ Department of Chemistry and Biochemistry, University of Oklahoma, Norman, Oklahoma 73019, United States; # Materials Science and Engineering, University of Colorado Boulder, Boulder, Colorado 80303, United States; ∇ Renewable and Sustainable Energy Institute, University of Colorado Boulder, Boulder, Colorado 80303, United States

## Abstract

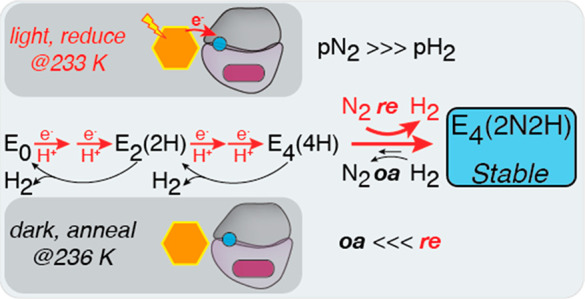

A critical step in the mechanism of N_2_ reduction to 2NH_3_ catalyzed by the enzyme nitrogenase is the reaction of the four-electron/four-proton reduced intermediate state of the active-site FeMo-cofactor (E_4_(4H)). This state is a junction in the catalytic mechanism, either relaxing by the reaction of a metal bound Fe-hydride with a proton forming H_2_ or going forward with N_2_ binding coupled to the reductive elimination (*re*) of two Fe-hydrides as H_2_ to form the E_4_(2N2H) state. E_4_(2N2H) can relax to E_4_(4H) by the oxidative addition (*oa*) of H_2_ and release of N_2_ or can be further reduced in a series of catalytic steps to release 2NH_3_. If the H_2_*re*/*oa* mechanism is correct, it requires that *oa* of H_2_ be associative with E_4_(2N2H). In this report, we have taken advantage of CdS quantum dots in complex with MoFe protein to achieve photodriven electron delivery in the frozen state, with cryo-annealing in the dark, to reveal details of the E-state species and to test the stability of E_4_(2N2H). Illumination of frozen CdS:MoFe protein complexes led to formation of a population of reduced intermediates. Electron paramagnetic resonance spectroscopy identified E-state signals including E_2_ and E_4_(2N2H), as well as signals suggesting the formation of E_6_ or E_8_. It is shown that in the frozen state when pN_2_ is much greater than pH_2_, the E_4_(2N2H) state is kinetically stable, with very limited forward or reverse reaction rates. These results establish that the *oa* of H_2_ to the E_4_(2N2H) state follows an associative reaction mechanism.

Nitrogenases are two-component enzyme systems that catalyze the ATP-dependent reduction of N_2_ to ammonia (NH_3_) and hydrogen (H_2_).^1^ Mo-nitrogenase is the most well-studied of the three nitrogenase systems (i.e., Mo-, V-, and Fe-nitrogenase), from which an understanding of the stepwise proton- and electron-transfer steps has evolved into a model of the N_2_ reduction scheme.^1−3^ The Fe protein component of MoFe-nitrogenase catalyzes the ATP-dependent delivery of electrons to the MoFe protein component,^4,5^ which harbors the iron–molybdenum cofactor (FeMo-co), which is the site of N_2_ binding and activation (Figure 1). Under ideal conditions, the catalytic cycle requires a minimum of eight electron-transfer steps from Fe protein to MoFe protein, producing two molecules of NH_3_ and one molecule of H_2_ from one N_2_, eight protons, and 16 ATP.^5,6^ By varying the reaction conditions (i.e., N_2_ partial pressure, [ATP], Fe protein:MoFe protein ratio, etc.) of nitrogenase reactions, intermediates have been trapped, leading to development of a kinetic model of the catalytic mechanism based on the Lowe–Thorneley (LT) scheme (Figure 1).^7−10^

**Figure 1:**
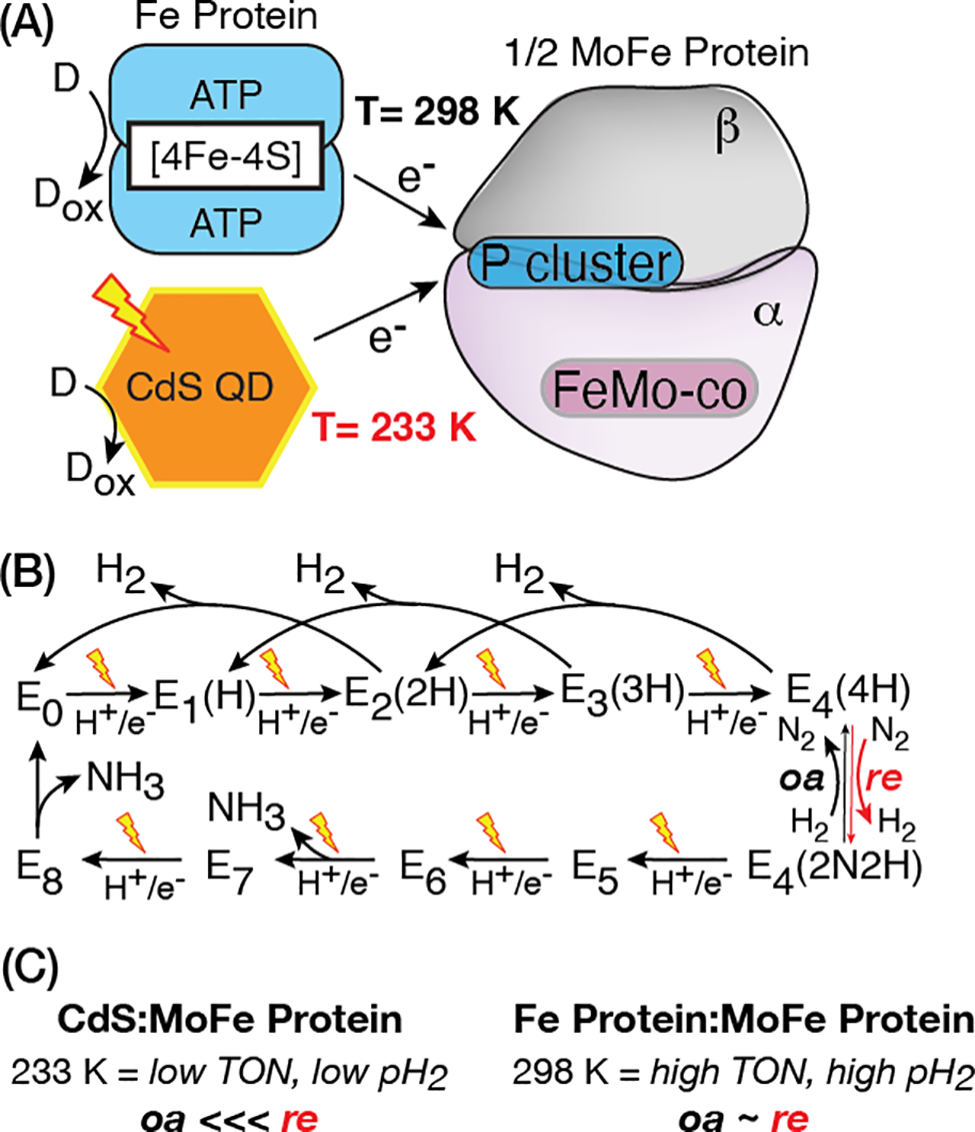
(A). Natural and biohybrid systems used for delivery of electrons to the nitrogenase MoFe protein. Electron delivery by Fe protein (blue) to MoFe protein (gray and purple) is coupled to repeated cycles of ATP binding and hydrolysis at 298 K. Photoexcited CdS quantum dots (orange) couple photon absorption to electron delivery, which can be performed across a range of ambient to subambient (i.e., 233 K) temperatures (D, donor; D_ox_, oxidized donor). (B). Modified Lowe–Thorneley scheme of the N_2_ reduction reaction; it is not the full scheme which includes N_2_ binding to E_3_.^7,13−15^ Stepwise proton and electron delivery to the MoFe protein FeMo-co site leads to reduction of E_0_ to form E_*n*_-states. E_4_(4H) binds N_2_ by reductive elimination (*re*) of H_2_ to form E_4_(2N2H). Kinetic stability of the E_4_(2N2H) intermediate is under control of the reaction product, H_2_, and E_4_(2N2H) can convert back to E_4_(4H) by oxidative addition (*oa*) of H_2_ and release of N_2_. (C). Photoexcited electron delivery by CdS in the frozen state under 1 atm of N_2_ (left) limits MoFe protein turnover (TON) and H_2_ production compared with ambient reaction temperatures with Fe protein (right).

A key aspect of the model for the N_2_ reduction reaction is that the resting state FeMo-co, E_0_, must be reduced by four electrons and four proteins to form E_4_(4H), the precursor to N_2_ binding and activation.^9−12^ A release of H_2_ from E_4_(4H) by reductive elimination (*re*) of two hydrides energetically drives the binding of N_2_ and formation of the E_4_(2N2H) intermediate (Figure 1).^11,12^ Therefore, the reversible conversion of E_4_(4H) to E_4_(2N2H) is a critical point in the catalytic mechanism between the forward pathway to NH_3_ formation and the backward pathway of H_2_ release. The reversible reaction involves the oxidative addition (*oa*) of H_2_ to E_4_(2N2H) that re-forms E_4_(4H). The dependence of *re*/*oa* kinetics on the pN_2_/pH_2_ ratio led to the prediction that E_4_(2N2H) is kinetically stable as N_2_ increases or if H_2_ decreases. Trapping reactions under different N_2_ partial pressures have been used to demonstrate that the decay of E_4_(2N2H) is slower as pN_2_ increases^6^ or when pH_2_ is lowered by flushing reactions under argon.^10,11^ Collectively, the results support that the pN_2_/pH_2_ ratio and the *re*/*oa* equilibrium control E_4_(2N2H) stability (Figure 1). However, H_2_ coproduction by nitrogenase under turnover prevents complete elimination of H_2_ and a determination of whether *oa* of H_2_ follows an associative versus dissociative process.

In earlier work, we demonstrated that MoFe protein can be reduced by photochemical electron delivery from photoexcited CdS nanocrystals (Figure 1).^16−20^ The physical coupling of nanocrystal materials and MoFe protein enables electron delivery under illumination at different temperatures.^17,20^ Illumination of reactions in the frozen state (∼233 K) has been used to photoaccumulate and trap P cluster or FeMo-co cluster intermediates in the absence of catalytic turnover.^18,19^ Therefore, this approach might be useful for generating and testing the stability of catalytic intermediates in the absence of turnover and H_2_ production. Herein, CdS quantum dot (QD)-MoFe protein biohybrids were prepared and illuminated in reactions at 233 K under 1 atm of N_2_, followed by cryo-annealing in the dark at 236 K (see Supporting Information for details). Changes in populations of even E-states (Figure 1) in MoFe protein under annealing can be monitored by electron paramagnetic resonance (EPR) spectroscopy for observing the relaxation kinetics of intermediates and analyzing the stability of the E_4_(2N2H) state.

Figure 2 displays the EPR spectra time course used to monitor the E-state signal intensities. *t* = 0 min corresponds to the end point of illumination, and the spectrum consists of a mixture of signals assigned to the E_0_ and E_2_ (*S* = 3/2), E_4_ (*S* = 1/2) intermediates (Tables S1 and S2).^10,11,21−24^ In addition, the simulation revealed signals consistent with those previously assigned to E_6_/E_8_ (Table S2). Annealing of the sample at 236 K in the dark, which prevents additional photoexcited electron transfer, led to some changes in signal intensities in the *S* = 3/2 and *S* = 1/2 regions of the EPR spectra (Figure 2). Simulations of each spectral time point were used to determine the E-state populations (Figure S1 and S2, Tables S3 and S4), which are plotted in Figure 3. Dark annealing led to a cumulative decrease in E_2_(2H)1b, E_2_(2H)1c, and E_4_(4H) attributed to the backward reaction, H_2_ release (Figure 1), which coincided with an increase in E_0_. Thus, the annealing of CdS:MoFe protein complexes at 236 K did not inhibit the hydride protonation at FeMo-co. In addition to these changes, signals matching those for the E_6_/E_8_ states also attenuated (Table S4).

**Figure 2:**
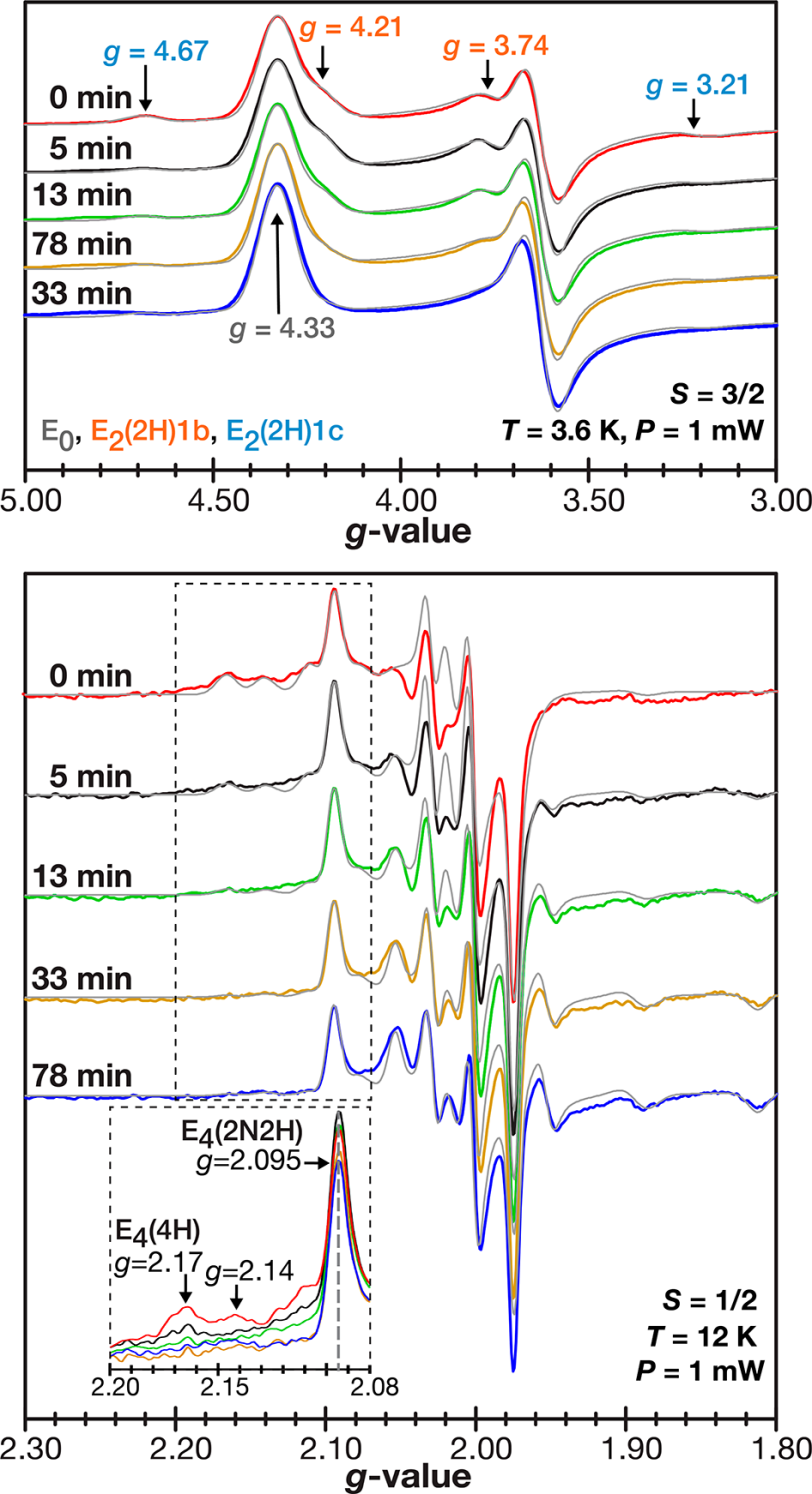
EPR spectra of the 236 K dark annealing time course. Top: The *S* = 3/2 region spectra (colored traces, *T* = 3.6 K, *P* = 1 mW) with callouts for *g*-values of E_0_, E_2_(2H)1b, and E_2_(2H)1c signals obtained from simulations (gray). Bottom: The *S* = 1/2 region spectra (colored traces, *T* = 12 K, *P* = 1 mW) with simulations (gray) (see Figure S2 and Table S2). The inset shows intensity changes of the *g*_*x*_ components of the E_4_(2N2H) (*g*_*x*_ = 2.095) and E_4_(H) (*g*_*x*_ = 2.17 and *g*_*x*_ = 2.14) signals. See the Supporting Information for experimental details, simulation methods, and simulated signals (Figures S1 and S2 and Table S1).

**Figure 3:**
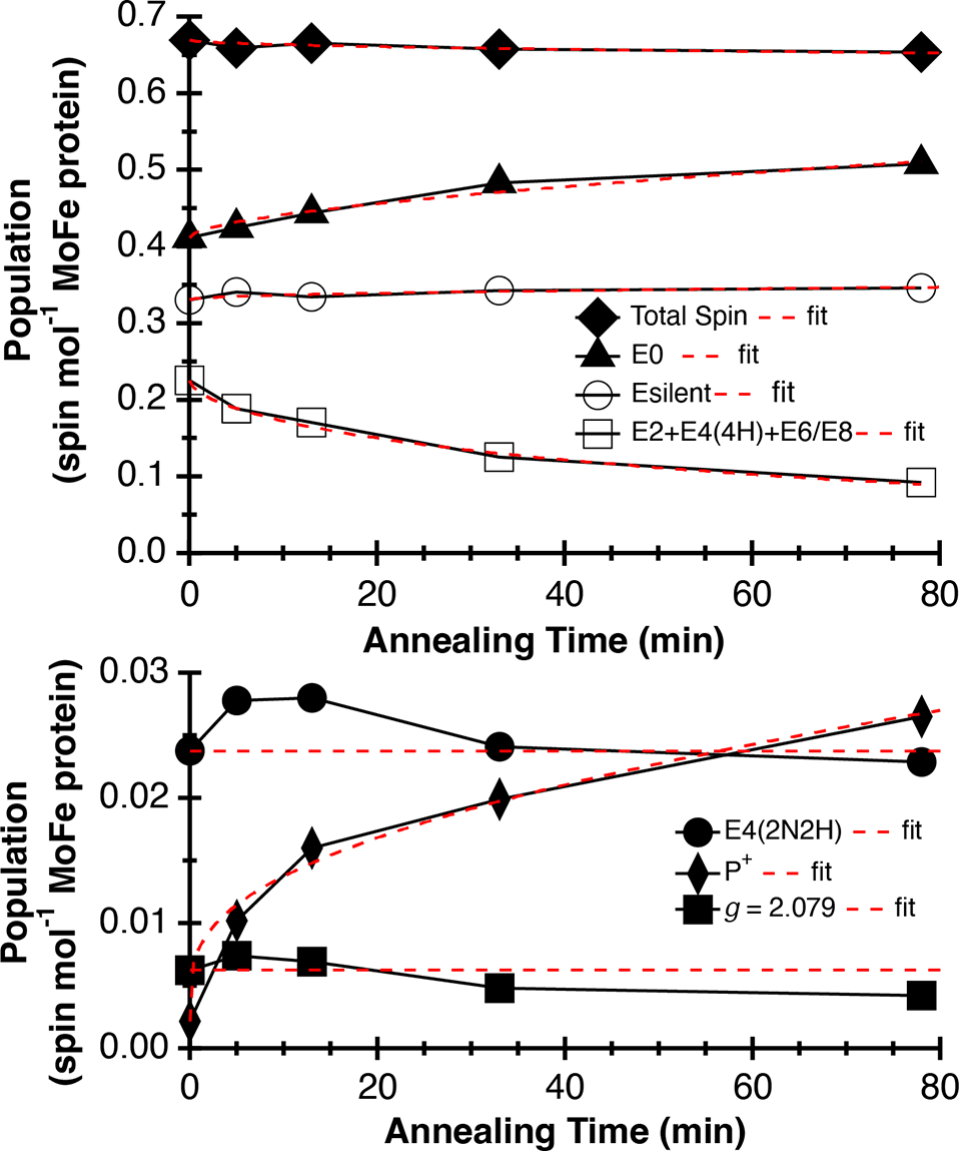
Time course of E-state populations during annealing at 236 K. The population values in spin mol^–1^ MoFe protein were obtained from simulations of EPR spectra at each annealing time point (Figure 2, Tables S3 and S4). Upper panel: Total spin (◆), E_0_ (▲), E_silent_ (○), E_2_+E_4_(4H)+E_6_/E_8_ (□). Lower panel: E_4_ (2NH2) (●). P^+^ (⧫), and *g* = 2.079 signal (■). Fits to eq 1 are shown as red dashed lines. Fit values are in Table S3; E_0_, τ = 1263 min, *m* = 0.55; E_silent_, τ = 4.6 × 10^4^ min, *m* = 0.47; E_2_ + E_4_(4H) + E_6_/E_8_, τ = 90 min, *m* = 0.61; E_4_(2N2H), τ = 3.6 × 10^6^ min, *m* = 0.98; P^+^, τ = 0.22, *m* = 0.15; *g* = 2.079, τ = 1.4 × 10^7^ min, *m* = 0.60.

To assess the decay kinetics of the E-states, the time course plots in Figure 3 were fit to the stretched exponential equation (eq 1):
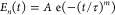
1where *E*_*n*_ (*t*) is the E-state population at time *t*, *A* is the value of *E*_*n*_(*t*) at *t* = 0, τ is the time constant for change in *E*_*n*_(*t*), and *m* is the breadth of the distribution, i.e., 0 < *m* ≤ 1 (fit values are in Table S5).^25,26^ The decline in the cumulative populations of E_2_, E_4_(4H), and E_6_/E_8_ (τ = 90 min) was ∼10-fold higher than accumulation of E_0_ (τ = 1263 min) (Figure 3, Tables S3 and S4), a difference that may result from other internal processes, such as P cluster oxidation (τ = 0.22) by electron transfer to FeMo-co that reduces the population of E_0_.

Most strikingly, there was no net loss in the E_4_(2N2H) population throughout the annealing time course (Figure 3). Under the conditions created here, there should be minimal formation of H_2_ from hydride protonation. Given that the rate of decay of the E_4_(2N2H) state back to the E_4_(4H) state is a second-order reaction, the combination of pN_2_ = 1 atm with a low pH_2_ (i.e., pN_2_≫pH_2_) is expected to result in a very low rate of *oa* (Figure 1). Consistent with this expectation is the observed slow decay of E_4_(2N2H), with a measured τ = 3.6 × 10^6^ min (Table S5).^10,11^ The net effect of the conditions created by the delivery of electrons to the MoFe protein in the frozen state using CdS nanoparticles is the stabilization of the E_4_(2N2H) state. These findings reveal that the *oa* reaction between E_4_(2N2H) and H_2_ is an associative rather than a dissociative process in which H_2_ binds to the E_4_(2N2H) state, rather than N_2_ being lost before H_2_ binds.

The understanding of the N_2_ reduction reaction by nitrogenases has largely developed from studies on the complete biological two-component system, where the requirement of ATP-dependent electron delivery by Fe protein places constraints on testing kinetic models in the absence of turnover. We have demonstrated that nanocrystals and light-controlled electron delivery can bypass some of these constraints to gain new insights into N_2_ reduction by nitrogenase. Here, CdS:MoFe protein biohybrids were used to clearly demonstrate the kinetic stability of the mechanistically central N_2_ bound state, E_4_(2N2H). The results give new insights into the nitrogenase mechanism, providing direct experimental evidence to support the assertion from theory that the E_4_(2N2H) state is stable at low concentrations of H_2_,^12^ revealing that the *oa* requires H_2_ binding to the E_4_(2N2H) state rather than release of N_2_ followed by H_2_ binding.

## Supporting Information Available

Materials and methods for CdS synthesis, MoFe protein isolation, EPR spectroscopy spectral simulations and spin quantifications; figures showing the *S* = 3/2 (*T* = 3.6 K, *P* = 1 mW) and *S* = 1/2 (*T* = 12 K, *P* = 1 mW) EPR spectra collected for CdS:MoFe protein for each dark annealing time step and spectral simulations; tables of EPR signal *g*-values and the values from fits to stretched exponential eq 1 (PDF)

## Abbreviations

CdS: cadmium sulfide
QD: quantum dot
MoFe protein: nitrogenase iron molybdenum protein
Fe protein: nitrogenase iron protein
FeMo-co: MoFe protein iron molybdenum cofactor
P cluster: MoFe protein [8Fe-7S] cluster
EPR: electron paramagnetic resonance
E_0_, E_2_, E_4_, etc.: catalytic intermediates of MoFe protein
*P*: power
*T*: temperature, *t*, time
atm: atmosphere
e^–^: electron
h^+^: hole
ATP: adenosine triphosphate
H: proton
H_2_: hydrogen gas
N_2_: nitrogen gas
NH_3_: ammonia
K: kelvin
h: hour
W: watt
mW: milliwatt
