# Supplementary material for: Cryo-annealing of Photoreduced CdS Quantum Dot–Nitrogenase MoFe Protein Complexes Reveals the Kinetic Stability of the E4(2N2H) Intermediate
Source: J Am Chem Soc. 2023 Sep 20;145(39):21165–9. doi: 10.1021/jacs.3c06832 (PMC10557137; doi:10.1021/jacs.3c06832)
Supplement: Supplementary file 1 — ja3c06832_si_001.pdf [file ja3c06832_si_001.pdf]

Supporting Information

Cryo-annealing of photoreduced CdS quantum dot–nitrogenase MoFe protein complexes reveals the kinetic stability of the E_4(2N2H) intermediate.

Gregory E. Vansuch[a,‡], David W. Mulder[a,‡], Bryant Chica[a], Jesse L. Ruzicka[b,‡], Zhi-Yong Yang[c], Lauren M. Pellows[b], Mark A. Willis[d], Katherine A. Brown[a], Lance C. Seefeldt[c], John W. Peters[d,e], Gordana Dukovic[b,f,g], and Paul W. King[a,g,*]

[a]Biosciences Center, National Renewable Energy Laboratory, Golden, CO 80401, United States. [b]Department of Chemistry, University of Colorado Boulder, Boulder, CO 80309, United States. [c]Department of Chemistry and Biochemistry, Utah State University, Logan, UT 84322, United States. [d]Institute of Biological Chemistry, Washington State University, Pullman, Washington 99163, United States. [e]Department of Chemistry and Biochemistry, University of Oklahoma, Norman, OK 73019, United States. [f]Materials Science and Engineering, University of Colorado Boulder, Boulder, CO 80303, United States. [g]Renewable and Sustainable Energy Institute (RASEI), University of Colorado Boulder, Boulder, CO 80303, United States.

Materials and methods

CdS quantum dot (QD) synthesis. All experiments were performed on CdS QDs synthesized and functionalized with 3-mercaptopropanoic acid (3-MPA, Strem Chemicals, ≥99%) as described and used in a previous study.[1] The concentration was determined using the absorption spectrum and sizing curves found in Yu et al.[2]

MoFe protein preparation. MoFe protein was prepared by expression in *Azotobacter vinelandii* (A.v.) and purified using nickel affinity chromatography as described previously.[3-4] Purified proteins were concentrated using a Millipore solvent-resistant stirred cell under an Ar atmosphere with appropriate molecular weight cutoff filters. MoFe protein purity, concentration, and activity were assessed as previously described.[5]

CdS:MoFe protein sample preparation. All chemicals were used as received, and all sample handling was performed in an anaerobic chamber (MBraun) under a N_2 atmosphere. The EPR sample was prepared as previously reported with a few modifications.[6] In summary, a 200 μL reaction was prepared in the MBraun chamber under 100% N_2 atmosphere that consisted of 43.1 μM MoFe protein and 43.1 μM CdS QDs in 50 mM HEPES pH 7.5 (at 298 K), 5 mM NaCl, ~5% glycerol by weight, 5 mM 3-MPA, and 5 mM sodium dithionite. Prior to preparation of the CdS:MoFe protein sample, the MoFe protein was buffer exchanged into the EPR reaction buffer using a 30 kDa centrifugal filter (Millipore) at least 4x for 10 min. The exchange was initially performed at between 6,000 and 14,000 x *g*, and any protein aggregation was re-suspended after gentle agitation by pipetting. Following sample preparation, the CdS:MoFe protein solution was loaded into a 4 mm outer-diameter quartz EPR tube and capped with a rubber septum. The tube was then removed from the anaerobic chamber and immediately frozen in liquid nitrogen. When not in use, the sample was stored uncapped in a liquid nitrogen dewar. When removed from storage, the frozen sample was transferred into the anaerobic chamber on dry ice, quickly capped under N_2 atmosphere, and then removed from the chamber and kept in liquid nitrogen. The integrity of the MoFe protein was verified by the EPR spectra at t = 0 min, which displayed a similar FeMo-co E_0 signal to the resting state spectra of Fe protein:MoFe protein complex, MoFe protein alone, and for CdS:MoFe protein complexes.[5]

CdS:MoFe protein sample illumination, dark annealing, and EPR spectroscopy. Illumination was conducted in an integrating sphere (Thor Labs), fitted with a computer controlled liquid nitrogen cryostat (ER 4131 VT, Bruker)[6] to maintain the sample at 233 K. The temperature of the cryostat was calibrated using a buffer solution and thermocouple probe. The sample was equilibrated to 233 K in the dark, and subsequently illuminated at 233 K under 2 W of 405 nm light (Thor Labs) for 2 1h periods for a total of ~ 2 h. Following illumination, the sample was removed from the integrating sphere and immediately submerged in liquid nitrogen. Outside of EPR measurements, the sample was stored in the dark in a liquid nitrogen dewar. Dark annealing was performed in the same integrating sphere with the temperature adjusted to 236 K. For these experiments, the CdS:MoFe protein reaction sample in the EPR tube was placed in the integrating sphere and annealed in the dark for a set time period (t = 5 min, 8 min, etc.), removed, and rapidly placed in liquid nitrogen to quench the annealing. After each annealing timepoint, the EPR spectrum was collected on a Bruker E-500 EPR spectrometer described previously.[5-6] The collection parameters and conditions were as follows; microwave power = 1 mW; modulation frequency = 100 kHz, modulation amplitude = 9.6 Gauss, and sample temperatures of 3.6 and 12 K. EPR spectra of buffer collected under identical conditions and parameters were used for background corrections. The integrating sphere temperature was calibrated before each annealing timepoint measurement.

EPR simulations and E-state population analysis. E-state populations over the annealing time course were derived from spectral simulation analysis. Spectral simulations at the individual timepoints were carried out in EasySpin[7] through the Matlab platform using the least-squares fit fitting function and the *g*-

strain parameter for line broadening. The quality of the fits was evaluated by overlay to experimental spectra (Figures S1 and S2). Signal assignments for the simulations were based on previously published FeMo-co and P-cluster signals identified in Fe protein:MoFe protein[8-17] and CdS:MoFe protein complexes.[5-6] S = 3/2 E_0 and E_2 signals were simulated from spectra at 3.6 K and 1 mW microwave power using pseudo S = ½ systems as described previously.[5] S = ½ E_4 signals were simulated from spectra at 12 K and 1 mW microwave power. The relative E-state populations were extracted from the overall simulation. For the ensuing spin analysis, the S = 3/2 signal of the sample prior to illumination (and any annealing) was assumed to represent 1 spin mol^{-1} MoFe protein. E_0 and E_2 species concentrations were determined by double integration of the individual signals and comparison to double integration of the total S = 3/2 signal prior to any illumination. E_4 signals were quantitated by comparison to a 75 μM copper (II) sample collected under non power saturating conditions (T = 12 K, P = 0.0005, 0.001, and 0.0025 mW), extrapolating for the difference in experimental microwave power condition (P = 1 mW) as described previously[5] and for differences in g-value by the Aasa-Vänngård method.[18] For comparison purposes, all spin concentrations are reported as spin mol^{-1} MoFe protein (Tables S3 and S4).

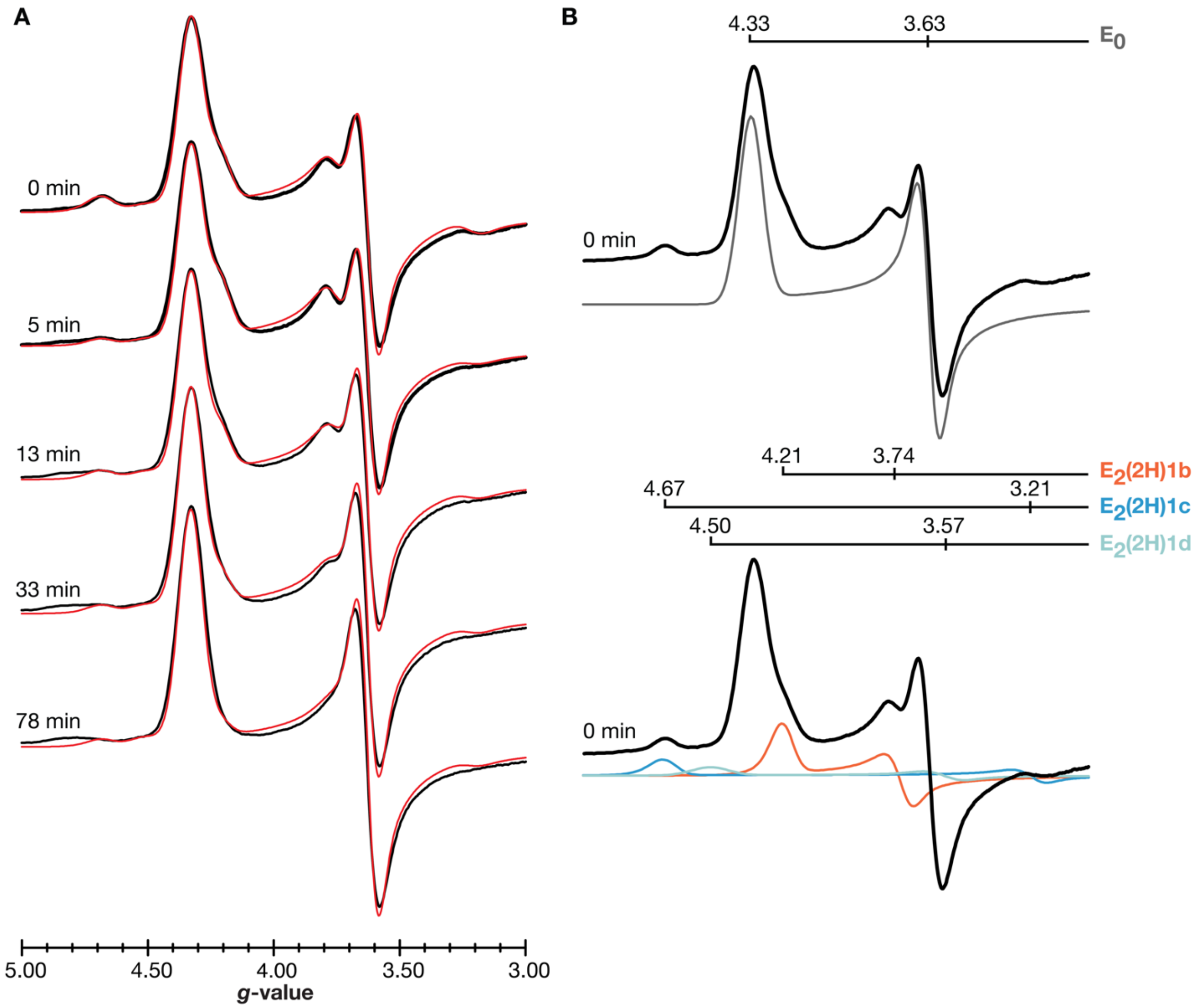

Figure S1. **A**. EPR annealing time series spectra of the $S = 3/2$ region (experimental spectra, colored lines; simulated spectra, red lines). $T = 3.6$ K, $P = 1$ mW. **B**. Simulation component breakdown for the $t = 0$ min annealing time point. The simulation components included E-type signals assigned to: **top**, E_0; and **bottom,** E_2(2H)1b, E_2(2H)1c, and E_2(2H)1d based on previous studies.[5, 10-11, 13] The origin of a broad signal at g = 4.8 which appeared during the annealing time course is unknown and was not simulated.

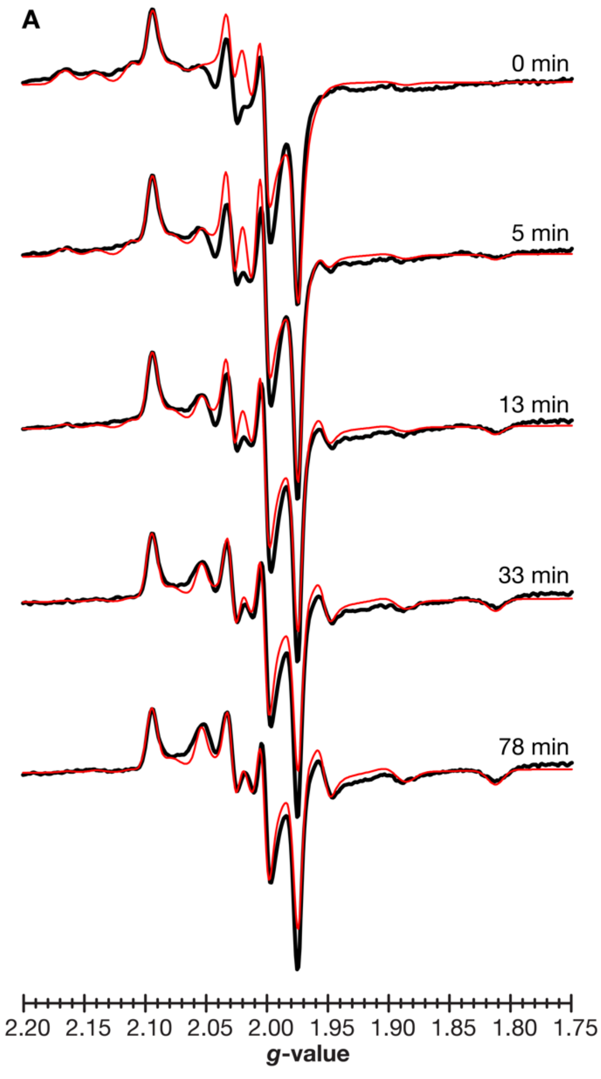

Figure S2. **A**. EPR annealing time series spectra of the S = ½ region (experimental spectra, black lines; simulated spectra, red lines). T = 12 K, P = 1 mW. **B**. Simulation component breakdown for the t = 0 min annealing time point. The simulation components were based on signals identified from previous studies[8-9, 12, 15-17] and included signals assigned to: **top**, E_4(4H) and E_4(2N2H) (green); **middle**, E_6/E_8 (purple) and an unknown signal at g = 2.079, 2.00, 1.975 (light purple); and **bottom**, E_0 (dark gray), P-cluster signals (orange) including $P^+_{1.81}$ (g = 2.05, 1.95, 1.81) and $P^+_{1.89}$ (g = 2.006, 1.89),[6, 19] and an isotropic signal at g = 2.03 (light gray).[6]

Table S1. EPR signals derived from simulations of S = 3/2 region for Fe protein:MoFe protein and CdS:MoFe protein complexes.

Reaction Complex	E-state signal g-values				Ref
	E_0	E_2(2H)1b	E_2(2H)1c	E_2(2H)1d	
Fe protein:MoFe protein	4.32, 3.66, 2.01	4.21, 3.76	4.69, 3.20	(4.58, 3.50, 1.99)	10-11, 13-14
CdS:MoFe protein	4.33, 3.63, 2.01	4.21, 3.74	4.67, 3.21	4.50, 3.57	5-6

Table S2. EPR signals derived from simulations of S = 1/2 region for Fe protein:MoFe protein and CdS:MoFe protein complexes.

Reaction Complex	E-state signal g-values						Ref
	E_4(4H)[a]	Unknown[b]	E_4(2N2H)[c]	E_6 or E_8[d]	$P^{+}_{1.81}$[e]	$P^{+}_{1.89}$[e]	
Fe protein:MoFe protein	2.15, 2.00, 1.96	N.A.	2.09, 1.99, 1.97	2.11, 2.01, 1.98<br>2.09, 2.01, 1.98	2.06, 1.95, 1.82	2.00, 1.89	8[a], 12[c], 15-17[d], 19[e]
CdS:MoFe protein	2.17, 2.01, 1.97<br>2.14, 2.01, 1.96	2.079, 2.00, 1.975	2.095, 2.00, 1.975	2.11, 2.00, 1.975<br>2.090, 2.00, 1.975	2.05, 1.95, 1.81	2.006, 1.89	This work, 6[e]

[b]N.A., not applicable.

Table S3. S = 3/2 E-state populations derived from simulations of EPR spectra at 236 K annealing timepoints.

Time (min)	E-state population (spin mol^{-1} MoFe protein)			
	E_0	E_2(2H)1b	E_2(2H)1c	E_2(2H)1d
0	0.41166	0.09697	0.05936	0.02372
5	0.42503	0.11097	0.02647	0.02117
13	0.44369	0.08274	0.03316	0.03574
33	0.48308	0.06006	0.03129	0.02643
78	0.50796	0.02887	0.02747	0.02887

Table S4. S = 1/2 E-state populations derived from simulations of EPR spectra at 236 K annealing timepoints.

Time (min)	E-state population (spin mol^{-1} MoFe protein)							
	E_4(4H) g = 2.17	E_4(4H) g = 2.14	E_4(2N2H)	E_6 or E_8 g = 2.09	E_6 or E_8 g = 2.11	Unknown g = 2.079	$P^{+}_{1.81}$	$P^{+}_{1.89}$
0	0.01376	0.01001	0.02377	0.00782	0.01376	0.00625	0.00156	0.00063
5	0.00788	0.00556	0.02781	0.00649	0.01019	0.00742	0.00927	0.00093
13	0.00426	0.00319	0.02799	0.00587	0.00587	0.00694	0.01494	0.00107
33	0.00121	0.00181	0.02410	0.00241	0.00242	0.00482	0.01809	0.00181
78	0.00121	0.00181	0.02290	0.00241	0.00180	0.00422	0.02412	0.00241

Table S5. Values for A, τ and m for each E-state population from fits to $E(t) = Ae(-(t/\tau)^m)$.

E-state	A	τ (min)	m
Etotal	0.6693	$7.7x10^4$	0.53
E_0	0.4113	1263	0.55
E_2 + E_4(4H) + E_6/E_8	0.225	90	0.61
Esilent	0.3307	$4.6x10^4$	0.47
E_4(2N2H)	0.0237	$3.6x10^6$	0.98
g = 2.079	0.0062	$1.4x10^7$	0.60
P^+	0.00218	0.22	0.15

References

1. Brown, K. A.; Ruzicka, J.; Kallas, H.; Chica, B.; Mulder, D. W.; Peters, J. W.; Seefeldt, L. C.; Dukovic, G.; King, P. W. Excitation-Rate Determines Product Stoichiometry in Photochemical Ammonia Production by CdS Quantum Dot-Nitrogenase MoFe Protein Complexes. *ACS Catal.* **2020,** *10*, 11147-11152.
2. Yu, W. W.; Qu, L.; Guo, W.; Peng, X. Experimental Determination of the Extinction Coefficient of CdTe, CdSe, and CdS Nanocrystals. *Chem. Mater.* **2003,** *15*, 2854-2860.
3. Christiansen, J.; Goodwin, P. J.; Lanzilotta, W. N.; Seefeldt, L. C.; Dean, D. R. Catalytic and Biophysical Properties of a Nitrogenase Apo-MoFe Protein Produced by a nifB-Deletion Mutant of *Azotobacter vinelandii*. *Biochemistry* **1998,** *37*, 12611-12623.
4. Jiménez-Vicente, E.; Del Campo, J. S. M.; Yang, Z.-Y.; Cash, V. L.; Dean, D. R.; Seefeldt, L. C., Application of Affinity Purification Methods for Analysis of the Nitrogenase System from *Azotobacter vinelandii*. In *Meth. Enzymol.*, Elsevier: 2018; Vol. 613, pp 231-255.
5. Chica, B.; Ruzicka, J.; Kallas, H.; Mulder, D. W.; Brown, K. A.; Peters, J. W.; Seefeldt, L. C.; Dukovic, G.; King, P. W. Defining Intermediates of Nitrogenase MoFe Protein During N_2 Reduction under Photochemical Electron Delivery from CdS Quantum Dots. *J. Am. Chem. Soc.* **2020,** *142*, 14324-14330.
6. Chica, B.; Ruzicka, J.; Pellows, L. M.; Kallas, H.; Kisgeropoulos, E.; Vansuch, G. E.; Mulder, D. W.; Brown, K. A.; Svedruzic, D.; Peters, J. W.; Dukovic, G.; Seefeldt, L. C.; King, P. W. Dissecting Electronic-Structural Transitions in the Nitrogenase MoFe Protein P-cluster During Reduction. *J. Am. Chem. Soc.* **2022,** *144*, 5708-5712.
7. Stoll, S.; Schweiger, A. Easyspin, a Comprehensive Software Package for Spectral Simulation and Analysis in EPR. *J. Magn. Reson.* **2006,** *178*, 42-55.
8. Lukoyanov, D.; Khadka, N.; Yang, Z.-Y.; Dean, D. R.; Seefeldt, L. C.; Hoffman, B. M. Reductive Elimination of H_2 Activates Nitrogenase to Reduce the N≡N Triple Bond: Characterization of the E4(4H) Janus Intermediate in Wild-Type Enzyme. *J. Am. Chem. Soc.* **2016,** *138*, 10674-10683.
9. Lukoyanov, D.; Yang, Z.-Y.; Khadka, N.; Dean, D. R.; Seefeldt, L. C.; Hoffman, B. M. Identification of a Key Catalytic Intermediate Demonstrates that Nitrogenase Is Activated by the Reversible Exchange of N_2 for H_2. *J. Am. Chem. Soc.* **2015,** *137*, 3610-3615.
10. Lukoyanov, D. A.; Khadka, N.; Yang, Z.-Y.; Dean, D. R.; Seefeldt, L. C.; Hoffman, B. M. Hydride Conformers of the Nitrogenase FeMo-cofactor Two-Electron Reduced State E2(2H), Assigned Using Cryogenic Intra Electron Paramagnetic Resonance Cavity Photolysis. *Inorg. Chem.* **2018,** *57*, 6847-6852.
11. Lukoyanov, D.; Yang, Z.-Y.; Duval, S.; Danyal, K.; Dean, D. R.; Seefeldt, L. C.; Hoffman, B. M. A Confirmation of the Quench-Cryoannealing Relaxation Protocol for Identifying Reduction States of Freeze-Trapped Nitrogenase Intermediates. *Inorg. Chem.* **2014,** *53*, 3688-3693.
12. Barney, B. M.; Lukoyanov, D.; Igarashi, R. Y.; Laryukhin, M.; Yang, T.-C.; Dean, D. R.; Hoffman, B. M.; Seefeldt, L. C. Trapping an Intermediate of Dinitrogen (N_2) Reduction on Nitrogenase. *Biochemistry* **2009,** *48*, 9094-9102.
13. Fisher, K.; Newton, W. E.; Lowe, D. J. Electron Paramagnetic Resonance Analysis of Different Azotobacter vinelandii Nitrogenase MoFe-Protein Conformations Generated During Enzyme Turnover: Evidence for S = 3/2 Spin States from Reduced MoFe-Protein Intermediates. *Biochemistry* **2001,** *40*, 3333-3339.
14. Maritano, S.; Fairhurst, S. A.; Eady, R. R. Novel EPR Signals Associated with FeMoco Centres of MoFe Protein in MgADP-Inhibited Turnover of Nitrogenase. *FEBS Letters* **2001,** *505*, 125-128.
15. Lukoyanov, D.; Barney, B. M.; Dean, D. R.; Seefeldt, L. C.; Hoffman, B. M. Connecting Nitrogenase Intermediates with the Kinetic Scheme for N_2 Reduction by a Relaxation Protocol and Identification of the N_2 Binding State. *Proc. Natl. Acad. Sci. U.S.A.* **2007,** *104*, 1451-1455.
16. Lukoyanov, D.; Dikanov, S. A.; Yang, Z.-Y.; Barney, B. M.; Samoilova, R. I.; Narasimhulu, K. V.; Dean, D. R.; Seefeldt, L. C.; Hoffman, B. M. ENDOR/HYSCORE Studies of the Common Intermediate Trapped During Nitrogenase Reduction of N_2H_2, CH_3N_2H, and N_2H_4 Support an Alternating Reaction Pathway for N_2 Reduction. *J. Am. Chem. Soc.* **2011,** *133*, 11655-11664.
17. Lukoyanov, D.; Yang, Z.-Y.; Barney, B. M.; Dean, D. R.; Seefeldt, L. C.; Hoffman, B. M. Unification of Reaction Pathway and Kinetic Scheme for N_2 Reduction Catalyzed by Nitrogenase. *Proc. Natl. Acad. Sci. U. S. A.* **2012,** *109*, 5583-5587.
18. Aasa, R.; Vänngård, T. EPR Signal Intensity and Powder Shapes: A Reexamination. *J. Magn. Reson. (1969)* **1975,** *19*, 308-315.
19. Tittsworth, R. C.; Hales, B. J. Detection of EPR Signals Assigned to the 1-Equiv-Oxidized P-clusters of the Nitrogenase MoFe-Protein from *Azotobacter vinelandii*. *J. Am. Chem. Soc.* **1993,** *115*, 9763-9767.
